# Supplementary material for: Multivariate associations between dopamine receptor availability and risky investment decision-making across adulthood
Source: Cereb Cortex Commun. 2023 May 15;4(2):tgad008. doi: 10.1093/texcom/tgad008 (PMC10225308; doi:10.1093/texcom/tgad008)
Supplement: dalearningCCA_supp_tgad008 [file dalearningcca_supp_tgad008.docx]

**Supplemental materials** for

Multivariate associations between dopamine receptor availability and risky investment decision making across adulthood

Mikella A. Green, Jennifer L. Crawford, Camelia M. Kuhnen, Gregory R. Samanez-Larkin, and Kendra L. Seaman

**Participants**

Thirty-seven healthy adults were recruited for a study of a large, multi-day, multimodal neuroimaging study of motivated cognition and decision making. Two participants were excluded (due to PET data quality issues), resulting in a final sample of 35. The age distribution is shown in Figure S1 below.


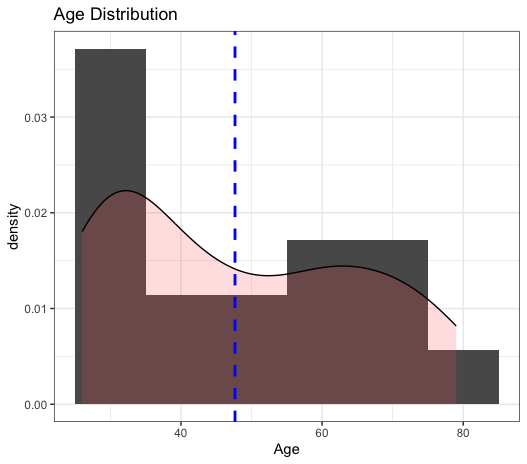


Figure S1. Age distribution of sample. Blue vertical line represents the sample mean.

**Behavioral Results**

This sample behaves similarly to prior samples (Figure S2; (Kuhnen, 2015), where participants demonstrate learning, but show deviations from optimal behavior. Participants have a bias towards picking stocks, as shown by the stock choices above 50% for most objective probabilities. Similar to prior studies (Kuhnen, 2015), participants overestimate the probability it is a good stock when the objective probability is low (less than 50%) and underestimate the probability it is a good stock when the objective probability is high (over 50%). Further, participants’ subjective estimates of probability are further away from Bayesian objective posterior estimates in the loss, compared to the gain, condition.


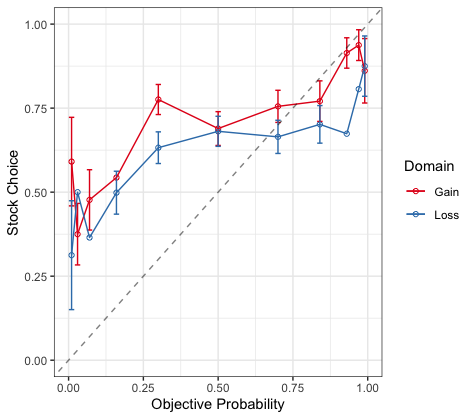

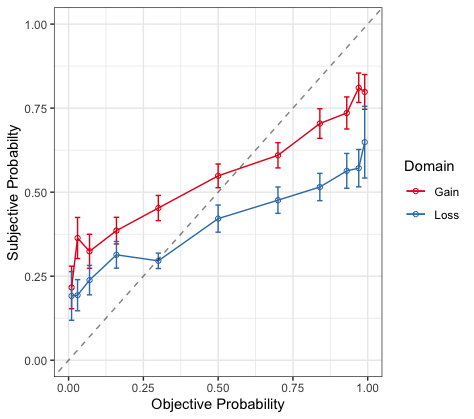


*Figure S2.* Stock-Bond Task Behavior. Group averages of (a) stock choices and (b) subjective estimates of probability that the stock is “good” on trials with the same Bayesian objective probability that the stock is “good”.

**Bivariate Correlations**

As seen in Table S1, age was moderately correlated with all brain variables (greater age was associated with lower D2 BPND) and choice inflexibility. All of the brain variables also correlated with each other, and two sets of behavioral variables correlated with each other.

| **Table S1** | | | | | | | | | | |
| --- | --- | --- | --- | --- | --- | --- | --- | --- | --- | --- |
| *Bivariate correlations between age, behavior, and dopamine receptor availability* | | | | | | | | | | |
| Variable | **1** | **2** | **3** | **4** | **5** | **6** | **7** | **8** | **9** |  |
| 1. Age | - |  |  |  |  |  |  |  |  |  |
| 2. Choice inflexibility | **0.39**  **[0.06, 0.64]** | - |  |  |  |  |  |  |  |  |
| 3. First choice stock | 0.18  [-0.17, 0.49] | 0.11  [-0.24, 0.43] | - |  |  |  |  |  |  |  |
| 4. Suboptimal investment | 0.27  [-0.08, 0.56] | **0.83**  **[0.69, 0.91]** | -0.18  [-0.49, 0.17] | - |  |  |  |  |  |  |
| 5. Probability estimation error | 0.11  [-0.24, 0.43] | 0.34  [0, 0.61] | -0.33  [-0.6, 0.01] | **0.54**  **[0.25, 0.74]** | - |  |  |  |  |  |
| 6. Optimism | -0.15  [-0.47, 0.2] | 0.2  [-0.15, 0.5] | -0.01  [-0.35, 0.33] | 0.16  [-0.19, 0.47] | -0.15  [-0.46, 0.2] | - |  |  |  |  |
| 7. Midbrain BP | **-0.64**  **[-0.81, -0.39]** | -0.14  [-0.45, 0.21] | -0.17  [-0.48, 0.18] | -0.1  [-0.43, 0.24] | 0.03  [-0.31, 0.37] | -0.08  [-0.41, 0.27] | - |  |  |  |
| 8. Amygdala BP | **-0.35**  **[-0.61, -0.01]** | **-0.35**  **[-0.62, -0.01]** | -0.26  [-0.55, 0.09] | -0.32  [-0.6, 0.02] | -0.13  [-0.45, 0.22] | 0.05  [-0.29, 0.38] | **0.65**  **[0.4, 0.81]** | - |  |  |
| 9. Insula BP | **-0.44**  **[-0.68, -0.12]** | -0.13  [-0.45, 0.21] | **-0.57**  **[-0.76, -0.29]** | -0.03  [-0.36, 0.31] | 0.07  [-0.27, 0.4] | 0.04  [-0.31, 0.37] | **0.51**  **[0.2, 0.72]** | **0.47**  **[0.15, 0.69]** | - |  |
| 10. ACC BP | **-0.48**  **[-0.7, -0.16]** | -0.1  [-0.42, 0.25] | **-0.38**  **[-0.64, -0.05]** | -0.02  [-0.36, 0.32] | 0.09  [-0.26, 0.41] | -0.19  [-0.5, 0.16] | **0.6**  **[0.33, 0.78]** | **0.46**  **[0.14, 0.69]** | **0.73**  **[0.52, 0.86]** |  |
|  |  |  |  |  |  |  |  |  |  |  |

**Alternate Canonical Correlation Analyses**

*Alternate analysis 1 – regress age out of all variables*

Per reviewer request, we regressed age out of all other variables and re-ran the CCA analysis to evaluate the relationship between dopamine receptor availability (controlling for age) and risky decision making (controlling for age). The results of this analysis were similar to the main analysis, albeit with one less function because there was one less predictor included (age). It produced four functions with squared canonical correlations (R^2^_c_) of 0.471, 0.352, 0.070, and 0.013. The full model was statistically significant using the Wilks’s lamba F-approximation, *F*(20, 87.2) = 1.82 *p* = .03. Dimension reduction analysis showed that the second through fourth functions did not explain statistically significant amounts of shared variance as seen in Table S2.

| **Table S2** | | | | | |
| --- | --- | --- | --- | --- | --- |
| Dimension reduction showing the significant shared variance overall but not in the second through fourth functions | | | | | |
| Roots | Wilks | F | Df1 | Df2 | p-value |
| 1 to 4 | 0.315 | 1.81 | 20 | 87.18 | 0.031 |
| 2 to 4 | 0.595 | 1.30 | 12 | 71.73 | 0.240 |
| 3 to 4 | 0.917 | 0.41 | 6 | 56 | 0.869 |
| 4 to 4 | 0.987 | 0.20 | 2 | 29 | 0.821 |

Table S3 shows the standardized canonical coefficients and structure coefficients for Function 1, along with the squared structure coefficients. As with the original analysis, the criterion variables suggest that choice inflexibility, suboptimal investment, and optimism are relevant contributors to the decision-making variate while the amygdala and midbrain are relevant contributors to the brain variate. The direction of these loadings is the same as the main analysis.

| **Table S3** |  | |  |  |
| --- | --- | --- | --- | --- |
| Standardized canonical coefficients (Coef), structure coefficients (r_s_), and squared structure coefficients (*r_s_^2^*) for each variable for Function 1. | | | | |
|  | | *Coef* | *r_s_* | *r_s_^2^* |
| *Brain variables* | |  |  |  |
| Midbrain | | 2.83 | 0.40 | 0.16 |
| Amygdala | | -1.29 | -0.42 | 0.17 |
| Insula | | -0.94 | -0.17 | 0.03 |
| Anterior cingulate | | 1.66 | 0.30 | 0.09 |
|  | |  |  |  |
| *Task variables* | |  |  |  |
| Choice inflexibility | | 7.42 | 0.61 | 0.38 |
| First choice stock | | 1.57 | 0.30 | 0.09 |
| Suboptimal investment | | 1.79 | 0.51 | 0.26 |
| Probability estimation error | | .006 | 0.35 | 0.12 |
| Optimism | | -0.054 | -0.52 | 0.27 |

*Alternate analysis 2 –* Use other brain regions as control

Per reviewer request, we re-ran CCA analyses with different ROIs to evaluate the relationship between dopamine receptor availability in alternate regions and risky decision making. The results of this analysis were different from the main analysis. It produced five functions with squared canonical correlations (R^2^_c_) of 0.502, 0.266, 0.238, 0.081, and 0.020. The full model was statistically significant using the Wilks’s lamba F-approximation, *F*(25, 90.7) = 1.64 *p* = .05. Dimension reduction analysis showed that the second through fifth functions did not explain statistically significant amounts of shared variance as seen in Table S4.

| **Table S4** | | | | | |
| --- | --- | --- | --- | --- | --- |
| Dimension reduction showing the significant shared variance overall but not in the second through fifth functions | | | | | |
| Roots | Wilks | F | Df1 | Df2 | p-value |
| 1 to 5 | 0.250 | 1.64 | 25 | 90.66 | 0.048 |
| 2 to 5 | 0.504 | 1.21 | 16 | 70.01 | 0.279 |
| 3 to 5 | 0.686 | 1.18 | 9 | 63.43 | 0.324 |
| 4 to 5 | 0.900 | 0.73 | 4 | 54 | 0.576 |
| 5 to 5 | 0.980 | 0.58 | 1 | 28 | 0.451 |

Table S5 shows the standardized canonical coefficients and structure coefficients for Function 1, along with the squared structure coefficients. Contrary to the original analysis, the criterion variables suggest that risky choice and optimism are relevant contributors to the decision-making variate while age, the frontal lobe and thalamus are relevant contributors to the age and brain variate.

| **Table S5** |  | |  |  |
| --- | --- | --- | --- | --- |
| Standardized canonical coefficients (Coef), structure coefficients (r_s_), and squared structure coefficients (*r_s_^2^*) for each variable for Function 1. | | | | |
|  | | *Coef* | *r_s_* | *r_s_^2^* |
| *Age and Brain variables* | |  |  |  |
| Age | | -0.02 | -0.28 | 0.08 |
| Temporal | | -1.72 | -.025 | 0.06 |
| Parietal | | 3.67 | -0.11 | 0.01 |
| Frontal | | -3.68 | -0.52 | 0.27 |
| Thalamus | | -0.02 | -0.37 | 0.13 |
|  | |  |  |  |
| *Task variables* | |  |  |  |
| Choice inflexibility | | -10.53 | -0.21 | 0.04 |
| First choice stock | | 4.22 | 0.60 | 0.36 |
| Suboptimal investment | | 3.72 | -0.19 | 0.04 |
| Probability estimation error | | 0.04 | 0.02 | 0.001 |
| Optimism | | -0.052 | 0.44 | 0.20 |

Although this conveys a slightly different picture, the effects are not as strong as those in the original analysis. The variables contributing to the brain variate are large, and thus not very informative. Further, the variables contributing to the behavioral variate are slightly different, with first stock choice, or risk taking, being the most dominate contributor to that variate. Despite this, we wanted to ensure that we were not overlooking something important and thus ran a third exploratory CCA to investigate which brain ROIs were important to include.

*Alternate analysis 3 –* Add alternate brain ROIs to original analysis

We ran a fourth CCA, this time with the four original ROIs (midbrain, amydala, insula, anterior cingulate), the four control (frontal, parietal, temporal and thalamus) ROIs, and behavioral variables. The results of this analysis were different from the main analysis. It produced five functions with squared canonical correlations (R^2^_c_) of 0.642, 0.527, 0.412, 0.222, and 0.146. The full model was statistically significant using the Wilks’s lamba F-approximation, *F*(45, 92.6) = 1.72 *p* = .015. Dimension reduction analysis showed that the second through fifth functions did not explain statistically significant amounts of shared variance as seen in Table S6.

| **Table S6** | | | | | |
| --- | --- | --- | --- | --- | --- |
| Dimension reduction showing the significant shared variance overall but not in the second through fifth functions | | | | | |
| Roots | Wilks | F | Df1 | Df2 | p-value |
| 1 to 5 | 0.066 | 1.72 | 45 | 92.57 | 0.014 |
| 2 to 5 | 0.185 | 1.43 | 32 | 79.04 | 0.101 |
| 3 to 5 | 0.391 | 1.17 | 21 | 63.72 | 0.303 |
| 4 to 5 | 0.665 | 0.87 | 12 | 46 | 0.583 |
| 5 to 5 | 0.854 | 0.82 | 5 | 24 | 0.549 |

Table S7 shows the standardized canonical coefficients and structure coefficients for Function 1, along with the squared structure coefficients. Similar to the original analysis, the criterion variables suggest that choice inflexibility, suboptimal investment, and optimism are relevant contributors to the decision-making variate while age and the amygdala BPnd are relevant contributors to the age and brain variate. Thus, the effects reported in the main text hold even when we add additional brain regions into the model.

| **Table S7** |  | |  |  |
| --- | --- | --- | --- | --- |
| Standardized canonical coefficients (Coef), structure coefficients (r_s_), and squared structure coefficients (*r_s_^2^*) for each variable for Function 1. | | | | |
|  | | *Coef* | *r_s_* | *r_s_^2^* |
| *Age and Brain variables* | |  |  |  |
| Age | | 0.05 | 0.61 | 0.37 |
| Midbrain | | 2.56 | -0.10 | 0.01 |
| Amygdala | | -1.00 | -0.45 | 0.20 |
| Insula | | -0.63 | -0.34 | 0.11 |
| Anterior cingulate | | -1.37 | -0.19 | 0.04 |
| Temporal | | 1.42 | -0.15 | 0.02 |
| Parietal | | -1.64 | -0.29 | 0.08 |
| Frontal | | 2.07 | -0.13 | 0.02 |
| Thalamus | | -0.20 | 0.004 | 0.00 |
|  | |  |  |  |
| *Task variables* | |  |  |  |
| Choice inflexibility | | 7.80 | 0.60 | 0.35 |
| First choice stock | | 0.12 | 0.14 | 0.02 |
| Suboptimal investment | | -0.69 | 0.51 | 0.26 |
| Probability estimation error | | 0.02 | 0.46 | 0.21 |
| Optimism | | -0.06 | -0.67 | 0.45 |
